# *In-vitro* antibacterial, antifungal, antioxidant and functional properties of *Bacillus amyloliquefaciens*

**DOI:** 10.1186/s12941-015-0069-1

**Published:** 2015-02-22

**Authors:** Shine Kadaikunnan, Thankappan Sarasam Rejiniemon, Jamal M Khaled, Naiyf S Alharbi, Ramzi Mothana

**Affiliations:** Department of Botany and Microbiology, College of Science, King Saud University, Post box 2455, Riyadh, 11451 Saudi Arabia; Department of Botany and Biotechnology, AJ College of Science and Technology, Thonnakal, Trivandrum, India; Department of Pharmacognosy and Medicinal, Aromatic & Poisonous Plants Research Center (MAPPRC), College of Pharmacy, King Saud University, PO Box 2457, Riyadh, 11451 Saudi Arabia

**Keywords:** *Bacillus amyloliquefaciens*, Antimicrobial activity, Probiotics, Antioxidant activities

## Abstract

**Background:**

Food born pathogenic bacteria and filamentous fungi are able to grow on most foods, including natural foods, processed foods, and fermented foods and create considerable economic loss. The aim of this study was to determine the antibacterial, antifungal, antioxidant and functional properties of *Bacillus amyloliquefaciens* recovered from silage.

**Methods:**

Minimum Inhibitory Concentration (MIC) of the compounds was assessed by using broth micro dilution method. The 1,1-diphenyl–2-picrylhydrazyl (DPPH)-radical scavenging and hydroxyl radical-scavenging abilities were measured to evaluate antioxidant activity of the strain.

**Results:**

Primary antimicrobial compound production screening revealed that *B. amyloliquefaciens* exhibited significant activity against all the tested bacteria and fungi compared to other strains. The 16S rRNA and gyrase A gene sequence analysis determined using molecular biological tools confirmed that the strain was 99% similarity towards *B. amyloliquefaciens*. The Minimum Inhibitory Concentration (MIC) of ethyl acetate extract against *Bacillus subtilis, Enterococcus cloacae* and *Staphylococcus aureus* were 25.0 μg ml^−1^, and *S, epidermidis* were 12.5 μg ml^−1^, respectively. Filamentous fungi *Aspergillus clavatus, A. fumigates, A. niger* and *Gibberella moniliformis* showed 25 μg ml^−1^. VJ-1 was able to survive the gastrointestinal conditions simulating the stomach and duodenum passage with the highest percentage of hydrophobicity. In addition, its resistance to hydrogen peroxide and highest hydroxyl radical and 2, 2-diphenyl–1-picrylhydrazyl (DPPH) scavenging activities, with inhibition rates of 56.84% and 67.12% respectively, were its advantage. An antimicrobial susceptibility pattern was an intrinsic feature of this strain, and thus, consumption does not represent a health risk to humans.

**Conclusion:**

*Bacillus amyloliquefaciens* might be a promising candidate for new pharmaceutical agents and probiotics.

## Introduction

Antimicrobial metabolites are widespread produced among bacteria of the genus *Streptomyces* and *Bacillus* [1]. In particular, *Bacillus* strains are ubiquitous in the environment; they have been isolated worldwide from soil, ocean, and organic matter respectively. Members of the *Bacillus* species are good sources of bioactive compounds, notably antibiotics, therapeutic proteins, enzyme inhibitors and pharmacologically active agents [2]. They produce a large number of antimicrobial metabolites and bio-peptides with different chemical structures and diversity, such as bacteriocins, bacteriocin-like substances and lipopeptides [3]. Although most of these substances are active against Gram-positive bacteria, some of them have a wide range of bio-activity towards Gram-negative bacteria and filamentous fungi [4]. Among antifungal compounds, lipopeptides identified to play a major role in disease suppression [5]. Different types of lipopeptides are produced by several *B. subtilis* and *B. amyloliquefaciens* strains. However; few lipopeptides such as surfactin, iturin and fengycin were recovered from *Bacillus* species and characterized [6-9]. Although a number of studies have been carried out on isolation and characterization of antimicrobial metabolite producing *Bacillus* from different origin, few assessments of their performance in anti-fungal studies have been reported. The objective of the present study was

- Isolation of *Bacillus amyloliquefaciens* VJ-1 from silage and molecular level identification by 16S rRNA and gyrase A (*gyrA*) gene sequencing
- Extraction of antimicrobial metabolites
- Evaluation of *in vitro* antibacterial and antifungal activities
- Evaluation of *in vitro* probiotic and antioxidant properties

## Materials and methods

### Chemicals and reagents

Culture media and antibiotics were purchased from Himedia, India. Dimethyl sulfoxide (DMSO) and 2, 2-diphenyl–1-picrylhydrazyl (DPPH) were obtained from Sigma-Aldrich. The genomic DNA isolation kit and pGEM-T vector were purchased from Promega (Madison, WI, USA).

### Isolation of *Bacillus* strains

The silage sample was used for the isolation of the new *Bacillus* strains. Briefly, an aliquot of 1 g of soil (sludge) sample was transferred into 100 ml of Luria-Bertani (LB, Himedia, Mumbai) medium (10 g l^−1^, casein peptone; 5 g l^−1^, yeast extract and 5 g l^−1^, NaCl; pH 7.0) and incubated aerobically at 37°C on an orbital shaker at 150 rpm for 24 h. After incubation the medium was serially diluted with saline (0.85% NaCl) and plated on LB agar. The plates were incubated at 37°C and observed after 24 h. Well isolated single colonies were selectively marked and propagated twice to check the purity and stored at −80°C with 20% sterile glycerol for further investigation and propagated twice in the same medium at 37°C before use. Antimicrobial activity of *Bacillus* strains were checked by growing the cells on Modified Nutrient Glucose Agar MNGA (10 g l^−1^, glucose; 5 g l^−1^, peptone; 3 g l^−1^, beef extract; 3 g l^−1^, dry yeast; 3 g l^−1^, NaCl and 3 g l^−1^, CaCO_3_) plates by single streak in the center [1]. Strain VJ-1, with very good antagonistic activity, was selected for further studies.

### Identification of *Bacillus* sp. VJ-1

#### Biochemical tests

The morphological properties were examined by light microscope. Biochemical and physiological properties of the isolate was analyzed using routine methods. API 50CHB test kit was used to characterize phenotypically. The API test strips were prepared according to the instructions of the kit supplier and scored after incubation for 24 h at 37°C.

### Antibiotic sensitivity and resistance pattern of *Bacillus* sp. VJ-1

Antibiotic sensitivity and resistance of *Bacillus* sp. VJ-1 was determined by disc diffusion method Arasu *et al.* [1]. Briefly, cells were prepared by growing in Muller Hinton (MH) medium (beef extract 2 g l^−1^, casein acid hydrolysate 17.5 g l^−1^, starch 1.5 g l^−1^, agar 17 g l^−1^) for 17 h at 37°C. Petri plates were prepared with 25 ml of sterile MH medium (Himedia). The test cultures (100 μl) of suspension containing 10^8^ CFU ml^−1^ bacteria) were swabbed on the top of the solid media and allowed to dry for 10 min. Different antibiotics loaded discs were placed on the surface of the medium and left for 30 min at room temperature for the diffusion of the antibiotics. Further, the plates were incubated for 17 h at 37°C. After incubation, *Bacillus* sp. VJ-1was classified as sensitive or resistant to an antibiotic according to the diameter of inhibition zone given in standard antibiotic disc chart.

### Scanning electron microscopy (SEM)

Twenty four h grown cells of *Bacillus* sp. VJ-1 were used for the SEM observations. The slats free cells were fixed by using glutaraldehyde (2.0%) and paraformaldehyde (2.0%) containing 0.1 M sodium cacodylate buffer (pH 7.2). After that the cells were coated with poly-L-lysine (75 mM). After dehydration cells were finally coated with gold at 30 mA for 150 sec and the picture was taken by SEM (Quanta™250FEG, FEI Company, Hillsboro, OR) at 12 kV.

### 16S rRNA and gyrase A (gyrA) gene polymerase chain reaction (PCR) and sequencing

The genomic DNA of *Bacillus* sp. VJ-1 was isolated by using kit method. 16S rRNA and gyrase A(*gyrA*) gene nucleotide sequences were amplified from chromosomal DNA by PCR using universal oligo-nucleotide primers, 27 forward primer (FP) (5′ AGA GTT TGA TCG TGG CTC AG 3′) and 1492 reverse primer (RP) (3′ GGT TAC CTT GTT ACG ACT T 5′) for (16S rDNA); 43 FP (5′ CAG TCA GGA AAT GCG TAC GTC CTT 3′), 1065 RP, (3′ CAAGGTAATG CTCCAGGC ATTGCT 5′) for (*gyrA*). The sequences were then compared in the GenBank database. Multiple sequence alignment was done and phylogenetic tree was constructed by the neighbor-joining method using MEGA (Version 4.1) software. The confidence level of each branch (1000 repeats) was tested by bootstrap analysis. The 16S rRNA sequences of *B. amyloliquefaciens* VJ-1was deposited in the GenBank database.

### Fermentation

#### Preparation of seed culture

To prepare the inoculum for fermentation experiments, two glycerol stock vials were used to inoculate 1000 ml flask containing 350 ml LB medium. The seed activation culture was grown at the 37°C for 16 h. Two generations of activation cultures were prepared before fermentation for transferring metabolically active cells. This culture was used as the seed culture.

### Batch fermentation of *Bacillus* sp. VJ-1 and extraction of metabolites

Batch fermentation was performed in a 10 l bioreactor (B-Braun). Seven liters of sterilized MNGA fermentation medium were added to the fermentor, and 350 ml (5%) seed culture was inoculated. Agitation was monitored at a low rate (200 rpm) just sufficient to mix the pH-control reagent into the medium. The fermentation temperature was adjusted to 37°C, and the pH was adjusted to 7.0 with 2 N NaOH. At the end of the fermentation cycle the culture was filtered and the supernatant was separated by centrifuging at 12,000 rpm for 20 min. The supernatant pH was adjusted to five by using 0.1 N HCl and then extracted with hexane, ethyl acetate and chloroform. The filtrates were concentrated under reduced pressure at 37°C and stored in a refrigerator at 4°C for subsequent antimicrobial screening. The percent yield of the extracts was 0.048, 0.029 and 0.067% (w/v) respectively for hexane, ethyl acetate and chloroform. The crude extracts were recuperated in DMSO and evaluated antimicrobial activity.

### Fungal biomass inhibition effect of *Bacillus* sp. VJ-1

Fungal biomass inhibition was examined by inoculating mid-log phase *Bacillus* sp. VJ-1into individual 250 ml Erlenmeyer flask containing 100 ml MNGA broth and cultivating them for 48 h at 37°C on an orbital incubator shaker. Cell-free supernatants were collected by centrifugation at 12,000 rpm for 20 min. Aliquots (10 ml) of supernatant with 40 ml of PD broth (potato 200 g l^−1^, dextrose 20 g l^−1^) were placed in 50 ml flasks and inoculated in triplicate with each test fungus. The fungal strains were incubated at 30°C for 5 days. Flasks without fermentation broth were the positive control. The growth performance of all fungal strains was checked separately in PD broth containing 100 mM acetic acid, lactic acid, ethanol and succinic acid to ensure that fungal inhibition was not simply due to nutrient exhaustion of the growth medium or acid production. After the incubation, fungal growth was measured by harvesting the cells, which were air-dried on preweighed Whatman #1 filter paper. Average fungal biomass was calculated for each test fungus and compared with the fungal biomass of positive controls.

### Minimum inhibitory concentration against bacteria

The minimum inhibitory concentrations (MIC) of extracts were tested against bacteria by broth micro dilution method [1]. The cell growth of the bacterial strains was adjusted to a McFarland standard 0.5 equivalent to concentrations of 1–5 × 10^8^ cfu/ml. The microbial suspensions were further diluted (1:100) in media to obtain a final inoculum of approximately 1.5 × 10^6^ cfu/ml. MIC was determined as the lowest concentration of the extracts inhibiting the visual growth of the test cultures. Three replicates were maintained to confirm the antifungal activity.

### Minimum inhibitory concentration against fungi

The antifungal activity and minimum inhibitory concentration (MIC) was performed according to the standard reference method [8]. Crude extracts were dissolved in sterile water together with 2% di-methyl sulfoxide (DMSO). The initial test concentration was serially diluted twofold. Each well was inoculated with 5 μl of suspension containing 10^4^ spore ml^−1^ of fungi. The antifungal agent ketoconazole was used as positive control. Plates were incubated 24, 48 or 72 h at 28°C. MIC was determined as the lowest concentration of the extracts inhibiting the visual growth of the test cultures. Three replications were maintained to confirm the antifungal activity.

### Microorganisms

Bacteria: *B. subtilis* (ATCC 7972), *Enterococcus cloacae* (ATCC 29212), *Staphylococcus aureus* (ATCC 25923), *S. epidermidis* (MTCC 3615). Fungi: *Aspergillus clavatus* (KCTC 40071), *A. fumigates* (KCTC 40080), *A. niger* (KCTC 40280), *A. oryzae* (KCTC 44823), *Curvularia lunata* (KCTC 40392), *Fusarium oxysporum* (KCTC 40051), *Gibberella moniliformis* (KCTC 44022), *Humicola grisea* (KCTC 40860), *Penicillium chrysogenum* (KCTC 40399) and *P. roqueforti* (KCTC 41354) were used for the experiment.

### Probiotic properties of *Bacillus* sp. VJ-1

#### Tolerance to low pH

Tolerance to low pH was determined using the plate count method. Briefly, active *Bacillus* sp. VJ-1 was grown in LB broth and was inoculated (1%) in 10 ml of fresh LB broth adjusted to pH 3.0 and 4.0 with hydrochloric acid (1.0 N) and incubated at 37°C for 3 h. Samples were withdrawn at 0 and 3 h of incubation to measure the initial bacterial population and residual cell population by plating suitable dilutions on LB agar plates. The plates were incubated at 37°C for 24 h, and the number of colonies that grew was counted. The experiment was performed in triplicate.

#### Bile tolerance

The ability of *Bacillus* sp. VJ-1 to grow in the presence of two different bile salts was studied according to the method of Vinderola and Reinheimer with slight modification [9]. LB-thiobroth (LB supplemented with 0.2% sodium thioglycollate) and LB-thiobroth supplemented with 0.3% (w/v) Oxgall were freshly prepared and inoculated overnight with 1% suspensions of *Bacillus* sp. VJ-1. Samples without Oxgall were considered the control. After 24 h incubation at 37°C, bacterial concentration was checked by a viable count determination on LB agar by plating suitable dilutions. The experiment was performed in triplicate.

#### Biogenic amine production

Production of biogenic amines was assessed by the method described by Bover-Cid [10]. Briefly, a 0.5 optical density at 600_nm_ (OD_600_) aliquot of freshly prepared *Bacillus* sp. VJ-1 cells were inoculated on LB agar medium supplemented with 1.0% each of lysine, tyrosine, ornithine, and histidine. Tween 80 (0.1%) was included in the medium to enhance bacterial growth. Bromocresol purple (0.006%) was used as the pH indicator. The formation of a clear purple halo was considered a positive reaction, indicating the presence of the respective amino acid decarboxylase. Medium without supplementing amino acids was considered the negative control, and the experiment was performed in triplicate.

#### Proteolytic activity

The proteolytic activity was measured by growing *Bacillus* sp. VJ-1 cells in 10% skim milk at 37°C for 42 h. The absorbance was read at 650 nm with an ELISA reader (Bio-Rad) [11]. The results were expressed as milligrams/milliliter tyrosine by means of reference to a calibration curve.

### Evaluation of cell surface hydrophobicity

The cell surface hydrophobicity assay was conducted according to the method described by Lee *et al.* with slight modifications [12]. Briefly, freshly prepared cells were centrifuged at 8000 rpm for 10 min. The cells were washed twice with PBS (pH 7.0). One ml of this suspension was used to determine the absorbance at OD_580_ nm. In duplicate assessments, a further 1 ml of this suspension was added to an equal volume of n-hexadecane (Sigma, USA) and was thoroughly mixed for 2 min using a vortex. The phases were allowed to separate at room temperature for 30 min, after which 1 ml of the upper phase was removed and the absorbance was determined at OD_580_nm. Percentage hydrophobicity was calculated as follows: (OD_580_ nm reading 1-OD_580_nm reading 2/OD_580_nm reading 1) × 100 = % hydrophobicity.

### *In vitro* antioxidant activity of *Bacillus* sp. VJ-1 cells

#### Resistance to hydrogen peroxide

The method of Buchmeier *et al.* was used with some modifications [13]. Briefly, 0.3 OD_600_ nm of *Bacillus* sp. VJ-1 cells were grown in 500 ml Erlenmeyer flasks containing 100 ml LB broth supplemented with 0.2, 0.4, 0.6, 0.8 and 1.0 mM hydrogen peroxide, at 37°C on an orbital incubator shaker. Cell growth was measured spectrophotometrically at 600_nm_, and increase in cell growth were measured as increases in optical density (OD).

#### Hydroxyl radical scavenging activity

The hydroxyl radical scavenging assay was conducted by a Fenton reaction method [14]. Briefly, the reaction mixture containing 1.0 ml of brilliant green (0.435 mM), 2.0 ml of FeSO_4_ (0.5 mM), 1.5 ml of H_2_O_2_ (3.0%, w/v), and 1.0, 1.5, 2.0 and 2.5 ml of freshly prepared *Bacillus* sp. VJ-1 cells cells (10^9^ CFU/ml) was incubated at room temperature for 15 min, and the absorbance was then measured at 624_nm_. The change in absorbance of the reaction mixture indicated the scavenging ability of the *B. amyloliquefaciens* VJ-1 cells for hydroxyl radicals.

$$ \mathrm{Scavenging}\ \mathrm{activity}\left(\%\right) = \left[\left({A}_s-{A}_0\right)/\ \left(A-{A}_0\right)\right]\kern0.5em \times \kern0.5em 100, $$

Where *A*s is the absorbance in the presence of the sample, *A*_0_ is the absorbance of the control in the absence of the sample, and A is the absorbance without the sample and Fenton reaction system.

#### DPPH free radical scavenging activity

The DPPH radical-scavenging capacity was determined according to the method described by Li *et al.* with some modifications [15]. Briefly, 1.0, 1.5, 2.0 and 2.5 ml of freshly prepared *Bacillus* sp. VJ-1 cells (10^9^ CFU/ml), was added to 1.0 ml methanolic DPPH radical solution (0.05 mM). The mixture was mixed vigorously and incubated at room temperature in the dark for 30 min. The controls included only deionized water and DPPH solution. The blanks contained only methanol and the cells. The absorbance of the resulting solution was measured in triplicate at 517_nm_, after centrifugation at 12000 rpm for 10 min.

$$ \mathrm{Scavenging}\ \mathrm{activity}\left(\%\right) = \left[1-\left({A}_{\mathrm{sample}}-{A}_{\mathrm{blank}}\right)/{A}_{\mathrm{control}}\right]\kern0.5em \times \kern0.5em 100. $$

## Results and discussion

### Isolation and identification of strain

**Figure 1:**
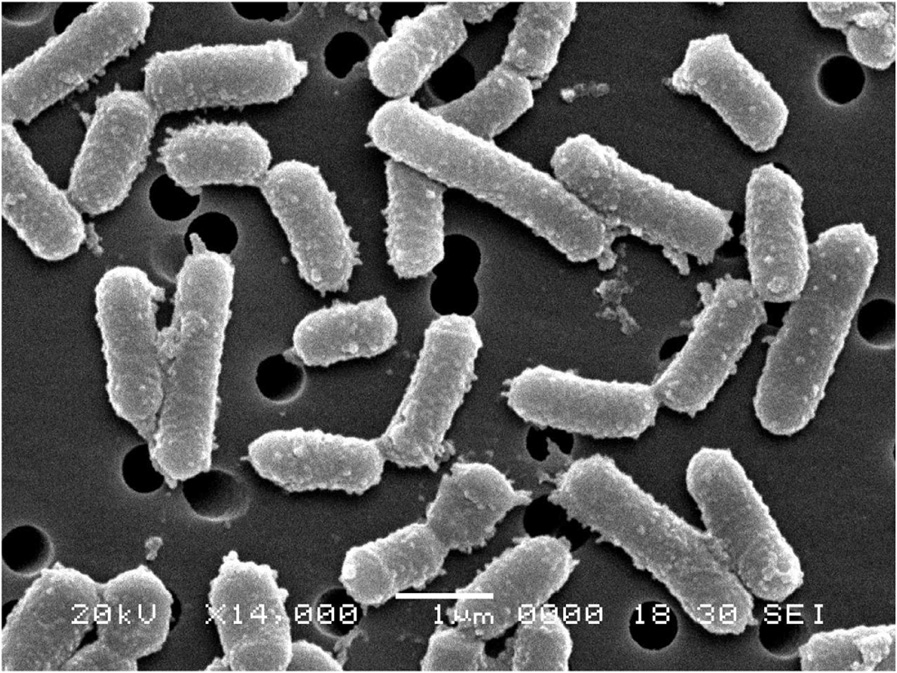
**Scanning electron microscopic image of** ***Bacillus amyloliquefaciens*** **VJ-1.**

**Figure 2:**
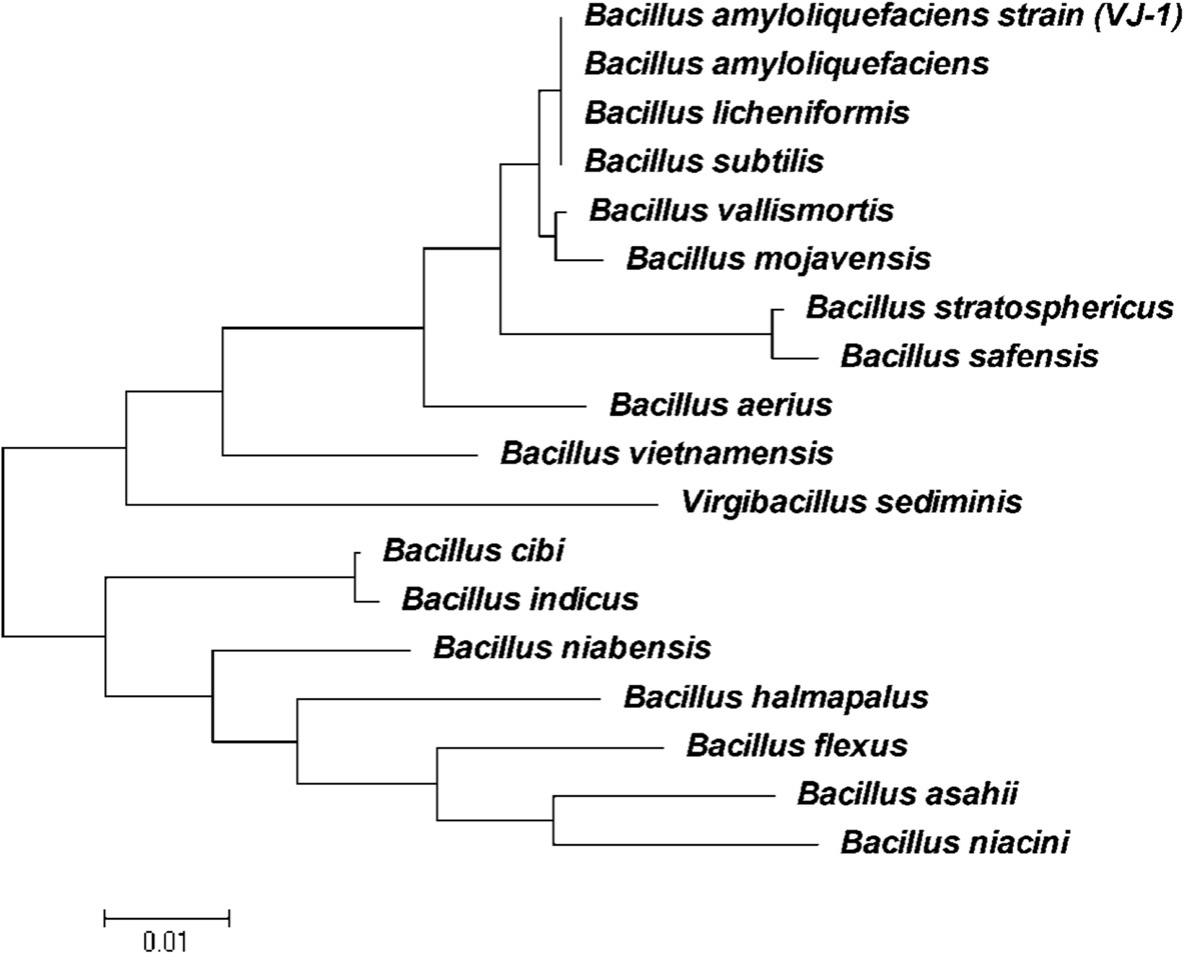
**Phylogenetic tree based on 16S rDNA gene sequence showing the relationship between VJ-1 strains and species belonging to the genus** ***Bacillus.*** The tree was constructed using the neighbor-joining method.

**Figure 3:**
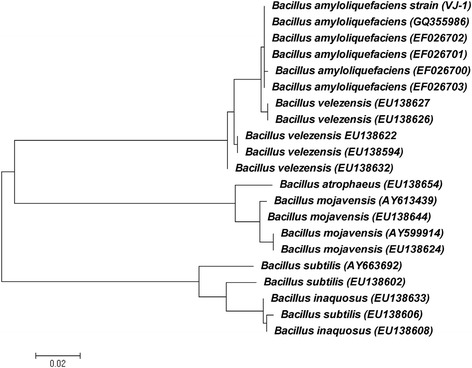
**Phylogenetic tree based on gyrase A gene sequence showing the relationship between VJ-1 strains and species belonging to the genus** ***Bacillus.*** The tree was constructed using the neighbor-joining method.

In the present study, 20 different strains were isolated from the silage samples, among which one strain named VJ-1 exhibited good antimicrobial activity against Gram positive and filamentous fungi were selected. The strain was observed as Gram-positive, aerobic, motile, rod-shaped (0.6-0.9 μm width and 1.9-3.10 μm length), and the SEM picture was shown in Figure 1. The morphology of the strain was tiny, opaque and cells were rough, leathery on the surface of the agar plates. Physiological and biochemical characters were firstly used to identify the strain after that subjected to identification by API system. The biochemical characters of the strain VJ-1 was showed in Table 1. Except for gentamicin (10 μg per disc), kanamycin (30 μg per disc), all the antibiotics tested in this study inhibited the growth of VJ-1 to some extent (Table 2). β-lactamase inhibiter group antibiotics ampicillin and imipenem showed the highest zone of inhibition 29 mm and 30 mm respectively. Most of the tested antibiotic disc documented above 20 mm as zone of inhibition. The results of biochemical and physiological characteristics revealed similar percentage of identity with *B. subtilis*. The percentage identity is to estimate of how closely the profile of the strain corresponds to the taxon relative to all other microorganisms. To confirm the strain, further identification was performed using 16S rRNA and gyrA gene amplification and sequencing. The 16S rRNA sequence of the strain showed that > 99% similarity of alignment to *B. subtilis*, *B. licheniformis*, *B. vallismortis*, *B. velezensis*, *B. mojavensis* and *B. amyloliquefaciens* (Figure 2). These species are considered as members of the *B. subtilis* group and their similarity on the basis of 16S rRNA sequence has been discussed [16]. However, 16S rRNA gene sequences showed limited variation in the closely related species of *B. subtilis* group (e.g. *B. subtilis* and *B. amyloliquefaciens* showed more than 99% similarities) and prevents the resolution of strain and species relationship [17]. Chun and Bae, reported that the sequence analysis of the gyrA sequence could accurately confirm *B. subtilis* and related taxa [16]. *Gyrase A* nucleotide sequences were analyzed by NCBI BLAST program showed the closest similarity relative to *B. amyloliquefaciens*, with sequence similarity of 99% (Figure 3). It is considered that, if the sequence identity showed above 99% similarity we could definitely conclude that the two strains belong to the same species, and if the identity is higher than 97%, strains are classified in the same genus or the same family. Therefore on the basis of morphological, physiological, biochemical characteristics, phylogenetic position and gene sequences of 16S rRNA and gyrA, the isolated strain was confirmed as *B. amyloliquefaciens*.

**Table 1:** **Biochemical and physiological characteristics of** ***Bacillus amyloliquefaciens*** **VJ-1**

**Substrates**	**Reactions/Enzyme**	**Results** ^**a**^	
		***B. amyloliquefaciens*** **VJ-1**	***B. subtilis*** ** (ATCC 7972)**
ONPG	β-galactosidase	-	-
Arginine	Arginine dihydrolase	-	-
Lysine	Lysine decarboxylase	-	-
Ornithine	Ornithine decarboxylase	+	+
Sodium citrate	Citrate utilization	+	+
Sodium thiosulfate	H_2_S production	-	-
Urea	Urease	-	-
Tryptophane	Tryptophane deaminase	-	-
Creatine sodium pyruvate	Acetoin production	-	-
Kohn’s gelatin	Gelatinase	+	+
Glucose	Fermentation/oxidation	+	+
Mannitol	Fermentation/oxidation	+	+
Inositol	Fermentation/oxidation	+	+
Sorbitol	Fermentation/oxidation	+	+
Rhamnose	Fermentation/oxidation	+	+
Sucrose	Fermentation/oxidation	+	+
Melibiose	Fermentation/oxidation	-	-
Amygdalin	Fermentation/oxidation	+	+
Arabinose	Fermentation/oxidation	+	+

+: Positive (more than 90%).

*−*: Negative (more than 90%).

^a^The results were measured after incubating the strains for 24 h at 37°C.

**Table 2:** **Comparative antibiotic sensitivity pattern of** ***Bacillus amyloliquefaciens*** **VJ-1 towards various antibiotics**

**Antibiotic group**	**Antimicrobial agent**	**Disc potency (** **μg)**	**Diameter of inhibition zone (mm)** ^**a**^
Aminoglycoside	Amikacin	30	22
	Gentamicin	10	0
	Kanamycin	30	0
	Streptomycin	10	11
	Tobramycin	10	25
Carboxypenicillin	Carbenicillin	50	27
β-lactamase inhibitor	Ampicillin	50	29
	Augmentin	30	25
	Imipenem	10	30
	Ticarcillin	75	31
Fluroquinolone	Ciprofloxacin	5	17
	Gatifloxacin	5	15
	Levofloxacin	5	20
	Moxifloxacin	5	20
	Nalidixic acid	30	22
	Norfloxacin	10	26
	Ofloxacin	5	26
	Sparfloxacin	5	28
Cephalosporin	Cefpodoxime	10	22
	Cetriaxone	30	25
Polymixin	Colistin	10	19
Sulphonamide	Co-Trimoxazole	25	18

^a^Zones of inhibition were measured after incubating the strains for 17 h at 37°C in LB agar medium.

### Fungal biomass inhibition effect

**Figure 4:**
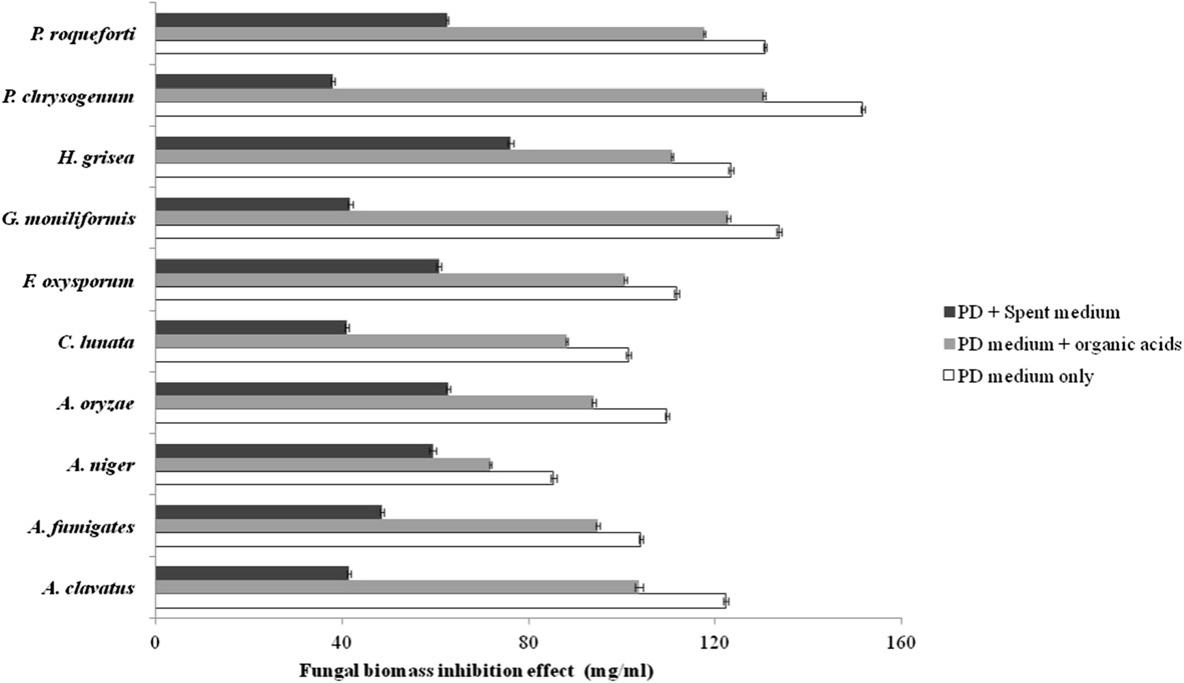
**Fungal biomass inhibition effect of** ***Bacillus amyloliquefaciens*** **VJ-1.** The fungal biomass inhibition effect of *Bacillus amyloliquefaciens* VJ-1 fermentation medium was evaluated by mixing with PD medium.

After three day incubation, VJ-1 significantly inhibited the growth of fungi, compared with that of LB controls and LB medium with lactic acid, acetic acid, succinic acid and ethanol based on dry weight measurements of fungal biomass (Figure 4). The greatest antifungal growth inhibitory activity of VJ-1 was recorded against *P. chrysogenum* (75.02%), followed by *G. moniliformis* (68.84%), *A. clavatus* (66.18%), *C. lunata* (59.56%) and *A. fumigates* (53.33%). The test fungi were not inhibited by the mixture of organic acids, suggesting that the fungal inhibition seen in the VJ-1 cell-free culture was due to the presence of other inhibitory compounds.

### Antibacterial activity

The antibacterial activities of the extracts were determined in terms of MIC (Table 3). Ethyl acetate and hexane extracts obtained from the isolates showed significant antimicrobial activity against Gram positive bacterial pathogens. Hexane extract exhibited MIC 50 μg/ml for *S. aureus*. It exhibited MIC value 75 μg/ml for *B. subtilis* and *E. cloacae* respectively. Whereas, *S. epidermidis* documented MIC value 100 μg/ml. The MIC values of the ethyl acetate extract are slightly similar or lesser than the standard broad spectrum antibiotic, streptomycin. The chloroform extract showed the highest MIC values. *B. amyloliquefaciens* GA1 produces cyclic peptides iturin A, surfactin and fengycin, and the iron-siderophore bacillibactin [18]. Iturin A was known for its antimicrobial activity. Marinomycin A isolated from *Marinispora* which inhibited the growth of human pathogenic bacteria such as methicillin-resistant *S. aureus* and vancomycin-resistant *E. faecium* [19].

**Table 3:** **Antibacterial activities of organic extracts obtained from** ***Bacillus amyloliquefaciens*** **VJ-1**

**Indicator bacteria**	**Inhibitory activity (mm)** ^**a**^	**Minimum inhibitory concentration (**μ**g/ml)**				
		**HE**	**EA**	**CE**	**C**	**S-1**
*Bacillus subtilis* (ATCC 7972)	20.16±0.28	75.00	25.00	125.00	12.50	7.50
*Enterococcus cloacae* (ATCC 29212)	19.00±0.5	75.00	25.00	125.00	12.50	6.25
*Staphylococcus aureus* (ATCC 25923)	22.33±0.57	50.00	25.00	100.00	25.00	6.25
*Staphylococcus epidermidis* (MTCC 3615)	29.83±0.76	100.00	12.50	100.00	31.50	3.75

HE: (Hexane extract), EE: (Ethyl acetate extract), CE: (Chloroform extract), S (Streptomycin), fungal control reference. Values are means of triplicate. ^a^The antibacterial activity were monitored by growing the bacteria in MH and incubated at 37°C for 17 h.

### Antifungal activity

Hexane, ethyl acetate and chloroform extracts were screened against fungi. Minimum inhibitory concentration values are reported in Table 4. All the fungi exhibited marked antagonistic activity against the extracts (ethyl acetate extract showed comparatively better activity than hexane and chloroform extracts). Ethyl acetate extracts exhibited MIC 2.5 mg/ml for *A. clavatus, A. fumigates, A. niger* and *G. moniliformis. A. oryzae* and *C.lunata* showed good activity 5.0 mg/ml. Overall the isolated extracts inhibited the growth of fungi higher to the standard antibiotic ketoconazole. There have already been many reports about the antifungal activities of metabolites isolated from the natural sources against *C. albicans, Cryptococcus neoformans, T. mentagrophytes* and *A. fumigates* [20]. The activity could be due to alteration in the cell membrane permeability [21]. It is known that some *B. amyloliquefaciens* strains could produce surfactin, iturin and fengycin production [22]. Iturins are a group of antifungal, cyclic lipopeptodes, consisting of iturin A-E, bacillomycin D, F and L, and mycosubtilin. There are several reports on antifungal activity of novel metabolites produced by microorganisms. Lavermicocca *et al.* reported that 3-phenyllactic acid produced by some strains of *Bacillus* exhibited growth inhibitory effects against a wide range of mould species, which includes *Aspergillus ochraceus*, *Penicillium roqueforti*, *P. citrinu* [23]. Sjogren *et al.* showed that 3-hydroxy fatty acids from *Bacillus* revealed significant antifungal activity against different molds and yeasts [24]. *Pseudomonas* sp. was known to produce phenazine-1-carboxylic acid, another antifungal secondary metabolite [25].

**Table 4:** **Antifungal activities of organic extracts obtained from** ***Bacillus amyloliquefaciens*** **VJ-1**

**Indicator fungi**	**Minimum inhibitory concentration (mg/mL)**			
	**HE**	**EA**	**CE**	**S-1**
*Aspergillus clavatus*	50.00	25.00	100.00	12.50
*Aspergillus fumigates*	50.00	25.00	100.00	25.00
*Aspergillus niger*	37.50	25.00	100.00	12.50
*Aspergillus oryzae*	25.00	50.00	>100	50.00
*Curvularia lunata*	50.00	50.00	>75	25.00
*Fusarium oxysporum*	25.00	37.50	100.00	12.50
*Gibberella moniliformis*	>100	25.00	100.00	25.00
*Humicola grisea*	>100	100.00	>100	37.50
*Penicillium chrysogenum*	>100	100.00	>100	37.50
*Penicillium roqueforti*	>100	100.00	>100	50.00

HE: (Hexane extract), EE: (Ethylacetate extract), CE: (Chloroform extract), S (Ketoconazole), fungal control reference. Values are means of triplicate. The antifungal activity were monitored by growing the fungi in PD medium and incubated at 30°C for 28 h.

### Antioxidant activity

#### Resistance to hydrogen peroxide

**Figure 5:**
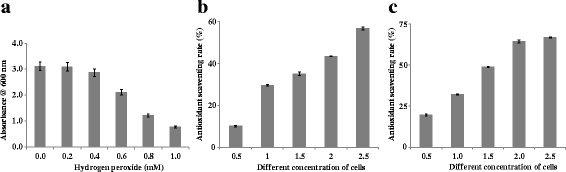
**Antioxidant activities of** ***Bacillus amyloliquefaciens*** **VJ-1. a**; Resistance at different hydrogen peroxide concentrations, **b**; scavenging activities on hydroxyl radicals, **c**; Scavenging effect on DPPH free radical.

The effect of hydrogen peroxide on the viability of the *strain* VJ-1 is shown in Figure 5(a). 4. Results revealed that the strain could able to tolerate 0.8 mM H_2_O_2_, whereas at 1.0 mM concentration the growth was slightly reduced with optical densities 0.76 after incubation for 8 h.

### Hydroxyl radical scavenging activity

The results for hydroxyl scavenging assay of the strain VJ-1 are shown in Figure 5b. The hydroxyl radical scavenging activity was increased with increasing quantity concentration the cells. The hydroxyl radical scavenging ability of the strain indirectly proportional to the concentration of the cells. The result revealed that the strain had the highest hydroxyl radical scavenging ability with an inhibition rate of 56.84% at 2.5 ml of cells at 10^8^ CFU/ml.

### DPPH free radical scavenging activity

The methanol extract of the strain VJ-1 exhibited a significant dose dependent inhibition of DPPH activity, with the highest radical-scavenging activity (67.33%) at 2.5 ml of cells at 10^8^ CFU/ml. The results are presented in Figure 5c.

### Probiotic properties

The ability of microorganisms to survive in the gastrointestinal tract condition is one of the main important characteristics required for probiotic bacteria. Therefore, the viability at low pH and bile salt conditions were studied. The viability of the VJ-1 at pH 3.0 and 4.0 are presented in Table 5. The result indicated that the cell viability was slightly increased at pH 3.0.Whereas, at pH 4.0 the strain withstands and grown. The survival ability of the strain in the presence of bile salts (Oxgall (0.3%) and sodium taurocholate (0.3%)) is presented in Table 6. The results revealed that the strain was able to grow in the presence of sodium taurocholate and was highly sensitive to oxgall. Proteolytic activity was determined as 0.051 mg/ml tyrosine liberation. A positive result for decarboxylase activity with tyrosine was observed, and exhibited high hydrophobicity (100%). Therefore strain VJ-1 is considered as safe in usage as probiotics as reported by the researchers [26-28].

**Table 5:** **Cell viability of** ***Bacillus amyloliquefaciens*** **VJ-1 under low pH**

**pH**	**0 min**	**30 min**	**60 min**	**90 min**	**120 min**	**150 min**	**180 min**
3.0	8.46±0.015	8.40±0.010	8.53±0.020	8.61±0.037	8.71±0.030	8.79±0.026	8.93±0.023
4.0	8.48±0.020	8.42±0.026	8.61±0.010	8.66±0.264	8.76±0.017	8.93±0.025	8.97±0.056

Values are means of triplicate determinations with standard deviations.

**Table 6:** **Cell viability of** ***Bacillus amyloliquefaciens*** **VJ-1 under bile salt condition**

**Sample**	**0 min**	**60 min**	**120 min**	**180 min**
Control	8.55±0.37	8.65±0.43	8.9±0.08	9.08±0.19
MRS+Na.Taurocholate	8.84±0.04	8.61±0.01	8.83±0.03	8.94±0.04
MRS+Na.Taurocholate+Oxgall	8.42±0.02	8.17±0.03	8.06±0.04	7.69±0.35

Values are means of triplicate determinations with standard deviations.

It has been suggested that probiotics play various biological roles through several mechanisms, one of the most debated being the antioxidant activity [29]. The antioxidant supplementation helps in reducing the level of oxidative stress and in slowing or preventing the development of complications associated with diseases [30]. It is reported that few probiotic strains such as *Streptococcus thermophilus*, *Bifidobacterium longum*, *L. plantarum* and *L. casei* possesses strong antioxidative activity, and are able to decrease the risk of accumulation of ROS during the ingestion of food [31]. The beneficial effects of probiotic strains with oxidative stress were reported by several authors [32-36]. DPPH free radical scavenging study indicated that the cell surface active compounds of *strain* VJ-1 may involved in the antioxidant activity and it is directly proportional to the cell concentration. The reason for the activity may because of the existence of enzymes, such as NADH-oxidase, SOD, NADH peroxide, and non-haem catalases respectively.

## Conclusions

From the present study, it is clear that a novel *B. amyloliquefaciens* VJ-1 produced extracellular product effective against bacteria and fungi. VJ-1 displayed significant biological activity against Gram positive bacterial pathogens and filamentous fungal pathogens. It contained good functional probiotic properties, such as high tolerance to low pH and bile salts. The strain exhibited strong hydrogen peroxide resistant ability, hydroxyl radical and DPPH free radical scavenging activity. Therefore, it is confirmed that this *B. amyloliquefaciens* VJ-1 strain possesses several suitable characteristics which make it appropriate for use with various probiotic products.
